# Multiple Comorbidity Profile of Psychiatric Disorders in Epilepsy

**DOI:** 10.3390/jcm10184104

**Published:** 2021-09-11

**Authors:** Agata M. Grzegorzewska, Mariusz S. Wiglusz, Jerzy Landowski, Katarzyna Jakuszkowiak-Wojten, Wiesław J. Cubała, Adam Włodarczyk, Joanna Szarmach

**Affiliations:** Department of Psychiatry, Faculty of Medicine, Medical University of Gdansk, 80-952 Gdansk, Poland; mwiglusz@gumed.edu.pl (M.S.W.); jland@gumed.edu.pl (J.L.); k.jakuszkowiak@gumed.edu.pl (K.J.-W.); cubala@gumed.edu.pl (W.J.C.); aswlodarczyk@gumed.edu.pl (A.W.); jszarmach@gumed.edu.pl (J.S.)

**Keywords:** anxiety disorders, depressive disorders, epilepsy, DSM-IV-TR (Diagnostic and Statistical Manual of Mental Disorders), SCID-I (Structured Clinical Interview)

## Abstract

The co-occurrence of psychiatric disorders in people with epilepsy (PWE) is not well documented or studied. Anxiety and depressive disorders are the most frequent comorbid disorders in PWE. In this paper, we characterized the rates of multiple psychiatric disorder comorbidity by reanalyzing data from a study sample of PWE. A total of 96 outpatient PWE completed the self-report symptom scale, and were diagnosed using the Structured Clinical Interview for Diagnostic and Statistical Manual of Mental Disorders (DSM-IV-TR) Axis I disorders (SCID-I). For analyses, patients were assigned to a comprehensive diagnostic group of anxiety and depressive disorders. In order to determine comorbidity across psychiatric diagnoses for the DSM-IV categories, Pearson’s chi-squared test (*χ*^2^) was used. In the study sample, eight patients (8.3% of the study sample, *n* = 96) had comorbid major depressive disorder and anxiety disorder. When looking at comorbidity of each diagnosis separately, it was determined that 50% of individuals with an anxiety disorder had comorbid Major Depressive Disorder (MDD) and 38% patients with MDD had comorbid anxiety disorder. This finding encourages a more systematic reporting of psychiatric prevalence data in epilepsy, especially taking into account the high ratio of multiple comorbid anxiety and depressive disorders in PWE.

## 1. Introduction

Patterns of comorbidity are of considerable interest in the general psychiatric literature but remain to be fully characterized among patients with epilepsy (PWE).

It is estimated that in the general population, 9.4% of people fulfil the criteria for an anxiety disorder and 8.2% fulfil the criteria for a depressive disorder in any 12-month period of time [1]. Several population-based studies have also reported elevated prevalence of anxiety and depressive disorders among PWE [2,3,4,5]. Recent findings suggest that the prevalence of anxiety and depressive disorders across studies on PWE are equivalent to the pooled prevalence of 20.2% (95% confidence interval (CI)—15.3–26.0%) and 22.9% (95% CI—18.2–28.4%), respectively [2]. However, depression and anxiety disorders in epilepsy are frequently reported as distinct disorders with little or no acknowledgement of their co-occurrence. In a meta-analysis of studies on prevalence of depressive and anxiety disorders in PWE, only three from 27 analyzed studies revealed data on co-occurrence of depressive and anxiety disorders [2].

Apart from in the general population, there is a paucity of accurate information on the prevalence of multiple psychiatric disorders in somatically ill populations, including PWE. When addressing psychiatric comorbidity in somatically ill patients, there is a tendency to recognize only single psychiatric identity, neglecting the possibility of multiple psychiatric comorbidities. In the general population, this tendency is now clearly recognized and is of major concern due to its possible negative impact on treatment outcomes in real-world settings. According to studies of the general population, between 10 and 20% of adults in any given 12-month period will visit their primary care physician while suffering from an anxiety or depressive disorder episode and more than 50% of these patients experience a comorbid second depressive or anxiety disorder [1,6,7,8,9,10]. International epidemiological and clinical studies reveal that comorbid depression and anxiety results in increased distress, impairment, and symptoms, resulting in a longer course than either of these disorders alone [11,12,13]. Due to the limited number of reports on their multiple comorbidity in epilepsy, the exact prevalence of comorbid anxiety and depressive disorders is still to be determined. Of note, there has been a lot of confusion about using term comorbidity, which has several different meanings in the literature, depending on the aim for which it is studied (e.g., as co-occurrence in clinical outcomes/mortality risk studies or to indicate a related medical condition). In psychiatry, it is often used in connection with nosological and/or etiological debates, usually to denote co-occurrence beyond that expected by chance alone. For the purposes of this report, comorbidity is defined broadly as the co-occurrence of anxiety, depressive disorders and epilepsy in the same person, regardless of the causal pathway linking them [14,15].

The aim of the study was to examine the rates of multiple comorbid psychiatric disorders by reanalyzing data from a study sample of PWE, with a particular focus on co-occurrence of mood and anxiety disorders in the study population.

## 2. Materials and Methods

### 2.1. Study Population

This study explores data collected as part of a registry reported elsewhere [16,17]. Briefly, 118 consecutive PWE from a tertiary epilepsy center were screened and 96 patients enlisted. Subjects included were those aged 18–65 who had been given a diagnosis of active epilepsy according to the International League Against Epilepsy criteria [18] and had been receiving stable antiepileptic treatment in the 2 months prior to study. The exclusion criteria were as follows: last seizure within 24 h of examination and more than 10 seizures in the last month (selected to minimize the impact of peri-ictal and ictal psychiatric symptoms), history of severe traumatic brain injury with midline shift as determined with neuroimaging, as well as neurosurgery, unstable disease or serious neurological disorder.

In addition, mental retardation, identification of psychogenic nonepileptic seizures (pseudo seizures), alcohol and/or drug dependence or abuse in the past 6 months and borderline/antisocial personality disorder, as determined by psychiatric interview, were considered as exclusion criteria, as the psychiatric set of symptoms present in those psychopathologic domains may confound evaluation of morbidity rates across anxiety disorders (ADs).

The study was performed in agreement with the Declaration of Helsinki, following the approval of the Ethic Research Committee of the Institution. For each study participant, written informed consent was obtained.

### 2.2. Evaluation

All subjects were assessed at a single study visit by the same investigator (MSW) and diagnosed with the Structured Clinical Interview for DSM-IV-TR Axis I Disorders (SCID-I) [19]. The structured interview was used to obtain information on disease history and sociodemographic status of patients that included age, gender, economic situation, marital status, age of seizure onset, seizure frequency, seizure type, experience of auras, duration of epilepsy, and duration of treatment, as well as psychiatric history and existence of lesions. Magnetic resonance/computed tomography imaging, electroencephalogram and laboratory test results were available for the majority of patients. Data were confirmed by referral source records from the epileptologist. For the purpose of this study’s analyses, patients were assigned to a comprehensive diagnostic group of depressive and anxiety disorders.

### 2.3. Statistics

In order to determine comorbidity across psychiatric diagnoses for the DSM-IV-TR categories, Pearson’s chi-squared test (*χ*^2^) was used. Frequencies and descriptive statistics were analyzed for each variable. Student’s *t*-tests for normally distributed continuous data, Mann-Whitney’s U-test for non-normally distributed data, and Fisher’s exact test for categorical data were used to make comparisons between groups. A value of *p* < 0.05 was considered to be statistically significant.

## 3. Results

The demographic characteristics of the study group are presented and discussed elsewhere [16,17]. In our study, we intend to focus on analyzing the rates of comorbid psychiatric disorders in PWE. In Table 1, clinical characteristics of patients with detailed analysis of the subgroups according to the presence of depressive disorders are presented.

According to the SCID-I, the diagnosis of any anxiety disorder was given in 16 (16.7%) patients, mainly panic disorder in 13 (13.5%) cases. Major depressive disorder was diagnosed in 21 (22%) patients, including a single episode in 17 (18%) cases. None of the patients had received a diagnosis of any psychiatric disorder before entering the study. The antiepileptic drugs (AEDs) used in the study group were carbamazepine (34.2%), sodium valproate (21%), lamotrigine (15.7%), and topiramate (8.5%). Subjects in the study group have not been given any other psychiatric or neurological medication apart from AEDs.

**Figure 1:**
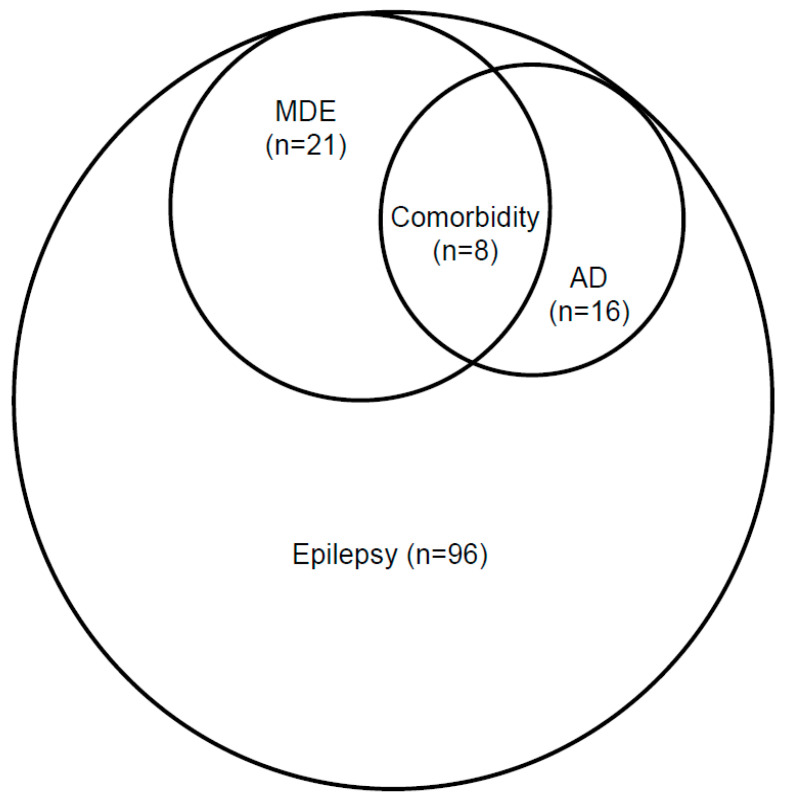
Comorbidity of Major Depressive Episode (MDE) with Anxiety Disorders (AD) in the group of epileptic patients (*n* = 96); Pearson’s test (χ^2^): χ^2^ = 8.8869, df = 1, *p* = 0.00287.

**Figure 2:**
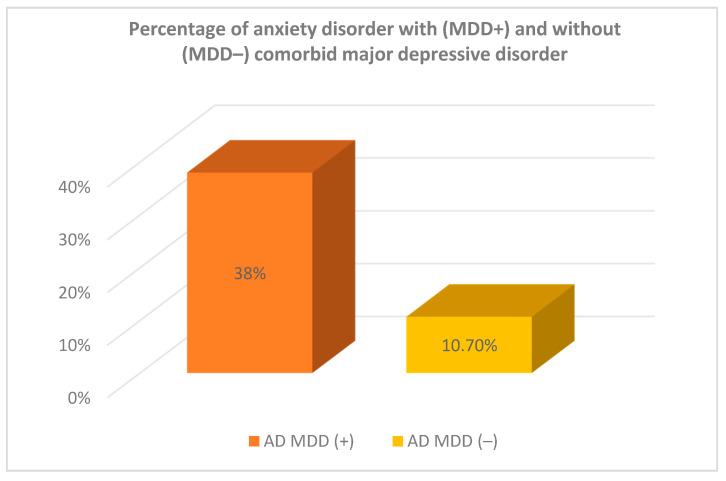

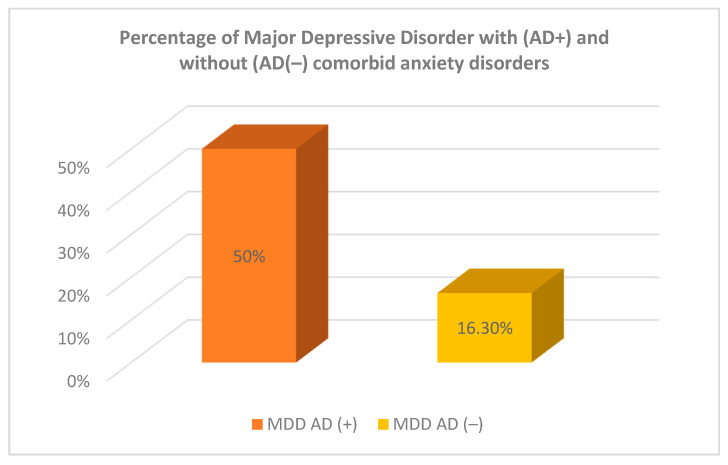
Percentage of patients with anxiety disorder/major depressive disorder with and without comorbid disorders. MDD = Major Depressive Disorder, AD = Anxiety Disorder, AD MDD(+) = Anxiety Disorder with comorbid Major Depressive Disorder, AD MDD(−) = Anxiety Disorder without comorbid Major Depressive Disorder, MDD AD(+) = Major Depressive Disorder with comorbid anxiety disorders, MDD AD(−) = Major Depressive Disorder without comorbid anxiety disorders.

In the study sample, eight patients (8.3% of the study sample, *n* = 96) had two comorbid disorders: major depressive disorder and anxiety disorder (Table 2, Figure 1). When looking at comorbidity of each diagnosis separately, it was determined that 50% individuals with anxiety disorder had comorbid MDD and 38% patients with MDD had comorbid anxiety disorder (Figure 2).

## 4. Discussion

This paper reports on clinically significant comorbidity between mood and anxiety disorders in patients with epilepsy, reaching 8.3% of the study sample.

The percentages of comorbidity differed when considering comorbidity profiles within each diagnosis separately, such that the proportion of individuals with a given disorder (AD) who also had a second disorder (50% of patients with AD suffering also from MDD) was not the same as the proportion of individuals with the second disorder (MDD) who also had the first disorder (38% of MDD subjects suffering from AD).

There has been little examination of the degree of comorbidity across psychiatric diagnoses in the epilepsy literature compared to studies on the general population. Our finding adds to the literature on this subject.

It is worth noting at this point that although we examined comorbidity rates for any anxiety disorder diagnosis, in the study sample, panic disorder (PD) was diagnosed in the majority of the patients, and that consisted of 81% of all anxiety disorder diagnoses.

In an excellent analysis of data on major depressive disorder and anxiety disorder comorbidity in the general population, the percentages of comorbidity differed across anxiety disorders [20,21,22]. For diagnostic pairs, the proportion of individuals with two comorbidities ranged from 4.5 to 20.3% within those diagnosed with at least one anxiety disorder. Remarkably, subjects diagnosed with PD had a high percentage of MDD comorbidity (51%). However those with MDD had a relatively lower percentage of AD comorbidity (20%) [20]. In our sample of PWE, similar rates were also observed when considering comorbid MDD in PD patients. What differed, though, was that the proportion of comorbid PD in MDD diagnosis was almost twice as large (38 vs. 20%) as in the general population in the study mentioned above [20]. The observed tendency raises the question of the frequency of panic disorder in PWE, which differs substantially across the studies, with recent data suggesting higher rates than reported earlier [17,23,24].

The high prevalence of anxiety disorders reported in the studies in PWE could have given rise to high comorbidity with depressive disorders. Unfortunately, due to a lack of reports on this subject, this possibility cannot be ruled out [2]. Gandy et al. [25,26] examined psychiatric comorbidity in a sample of 147 adult epilepsy outpatients, according to DSM-IV criteria, using the Mini International Neuropsychiatric Interview (MINI). Anxiety disorders were present in 30% of the sample (general anxiety disorder—30%, agoraphobia—10%, panic disorder with agoraphobia—2%, social phobia—5%, obsessive-compulsive disorder—0.7%), with 6% of participants meeting criteria for two or more anxiety disorders. Depressive disorders were present in 31% of the sample (MDD—24%, dysthymia—7%). A co-occurrence of anxiety and depressive disorder was present in 16% of the sample. In another study, psychiatric diagnosis criteria were established in 188 consecutive PWE with the MINI [27]. In that study group, 28 (15%) patients had current (1 or >1 type) anxiety disorder (general anxiety disorder—11%, agoraphobia—15%, panic disorder—2%, social phobia—9%, obsessive-compulsive disorder—3%). Current mood disorder (MDD plus dysthymia) was present in 31 (16%) patients (MDD—16%, dysthymia—3%). High comorbid occurrence of mood and anxiety disorders in PWE is illustrated by the data of the study. Most subjects with major depressive episodes suffered from comorbid anxiety disorder (*n* = 12 [11%]), and two-thirds of those patients fulfilled the criteria of DSM-IV for more than one type of such disorders. 

Finally, Jones et al. [5] interviewed 174 PWE from tertiary medical centers using the mood disorders module of the SCID and the MINI. Axis I disorders were apparent in the sample with anxiety disorders prevailing (total—52.15%: agoraphobia—15.5%, general anxiety disorder—13.2%, social phobia—10.9%, panic disorder—3.4%, obsessive-compulsive disorder—3.4%) and depressive disorders with a total of 21.2% (MDD—17.2%, dysthymia—4.0%). Recently, in a cross-sectional study on a Thai population, a total of 170 PWE from outpatient clinics was evaluated according to MINI for DSM-IV [28], among which 43 (25.3%) fulfilled the diagnostic criteria for one or more psychiatric disorders (mood disorders—17.1%, psychotic disorders—8.2%, anxiety disorders—5.3%, obsessive-compulsive disorder—2.9%). Eleven (6.5%) patients met the criteria for two or more mental disorders.

If taken into account that anxiety disorders and their comorbidity with depressive disorders amongst PWE have undergone relatively little research and received little clinical attention, it may be that clinicians did not assess for anxiety disorders or/and other comorbid psychiatric conditions in PWE as comprehensively as they did for depressive disorders [2]. In addition, some anxiety symptoms may be confused with seizure phenomena (including ictal fear that could be mistaken for panic attack) [29,30], or with side effects of AEDs, which could confound the study results. For example, it has been argued that many PWE experience atypical depressive symptoms, which contain overlapping anxiety and depression symptoms commonly referred to as interictal dysphoric disorder (IDD) [31].

Symptoms of depression or anxiety can be a part of peri-ictal dysphoric disorder (PDD). A cross-sectional study in tertiary referral centers in Europe indicated that around 13% of patients may experience depressed mood, dysphoria or irritability prior to seizures [32].

In a case series study of presurgical patients, up to 18% postictally reported at least five symptoms of depression that lasted more than 24 h, up to 22% developed symptoms of mania often accompanied by hallucinations or delusions [33] and 45% reported postictal anxiety [33]. However, we also cannot rule out that the observed number of overlapping symptoms in IDD is affected by the grades of comorbidity between depression and anxiety disorders or in fact could be better described by a multiple comorbidity pattern in PWE. This hypothesis could challenge a widely held assumption that psychiatric disorders in PWE have mostly atypical characteristics and therefore cannot be adequately described by means of current psychiatric classifications. This rationale is adequate, as multiple diagnoses commonly co-occur. Multimorbidity patterns are well known in medicine, especially in older people, amongst whom multiple concurrent chronic conditions are quite common [34,35]. It is well known that multimorbidity generates synergetic negative effects, and studies were employed to determine multimorbid disease clusters [34,35,36]. For example, a mental health cluster consists of: mood disorder, anxiety disorder, migraine headaches and/or thyroid disorder.

The awareness of psychiatric comorbidities in PWE could also could help us understand predictive factors of resilience and vulnerability in PWE [37], especially that the comorbid occurrence of mood and anxiety disorders has an even worse negative impact on the quality of life of PWE than anxiety or depressive disorders alone [27]. This could also lead to the development of psychological interventions aimed at strengthening resilience and coping strategies in PWE.

The understanding of multiple psychiatric comorbidities in epilepsy is an important area for future research to address and is crucial in order to improve the treatment of psychiatric comorbidities and epilepsy simultaneously.

## 5. Study Limitations

This is a post hoc analysis of a PWE sample with no control group with healthy individuals that may represent the general population. Thus, this study’s results refer to the PWE group and must not be applied to the general population.

Another study limitation is the small size of the PWE sample and potential selection bias, as the tertiary reference center was associated with a risk of complicated course of epilepsy. Subjects who had experienced more than 10 seizures in the last month and with the last seizure within 24 h of examination were excluded, but still the clinical manifestation of anxiety disorder may be confounded with seizure phenomena. Another limitation could be the occurrence of either ictal or postictal anxiety, which could be experienced within 72 h of a seizure and should be differentiated from the diagnosis of anxiety disorder. The factors mentioned above were partially excluded at the clinical interview as confounding. As our study is a type of correrational and observational study, it may provide susceptibility to bias and could be less precise, since it is not based on cause and effect analysis of the accessible variables.

## 6. Conclusions

In this study, sample eight patients (8.3% of the study sample, *n* = 96) had comorbid major depressive disorder and anxiety disorder. When looking at the comorbidity of each diagnosis separately, it was determined that 50% of individuals with AD had comorbid MDD and 38% patients with MDD had comorbid AD. This highlights the importance of the systematic and careful diagnostic approach in order to improve our understanding of multiple psychiatric comorbidities in PWE.

## Figures and Tables

**Table 1:** Clinical characteristics of subgroups according to the presence of depressive disorders.

	No Affective Disorders (*n* = 56)	MDD (*n* = 21)	Other Depressive Disorders (*n* = 19)	*p*
Epilepsy-related characteristics				
Age of seizure onset: mean (SD)	16.8 (10.3)	27.4 (13.5) *	18.5 (8.8)	0.001 ^#^
Duration of epilepsy: mean (SD)	18.5 (11.1)	16.8 (12.6)	12.9 (9.1)	
Number of seizures/last month-median (IQR)	4 (2, 5)	3 (2, 6)	2 (2, 8)	
Seizure type (%)	3 (5.4)	1 (4.8)	3(15.8)	
simple partial	16 (28.6)	5 (23.8)	6 (31.6)	
complex partial	26 (46.4)	13 (61.9)	8 (42.1)	
partial evolving to general	8 (14.3)	0 (0.0)	2 (10.5)	
tonic–clonic	2 (3.6)	0 (0.0)	0 (0.0)	
absence	0(0.0)	1(4.8)	0 (0.0)	
myoclonic atonic	1 (1.8)	1 (4.8)	0 (0.0)	
Number of AEDs (IQR)	1.5 (1, 2)	1 (1, 2)	2 (1, 2)	
Receiving AEDs with negative psychotropic effects (%)	16 (28.6)	5 (23.8)	8 (42.1)	
Drug resistant (%)	43 (76.8)	14 (66.7)	13 (68.4)	
Polytherapy (%)	26 (46.4)	8 (38.1)	12 (63.2)	

^#^ one-way ANOVA; * MDD vs. no affective disorders, *p* = 0.0005; MDD vs. other depressive disorders, *p* = 0.0201 (*t*-Student test). MDD—Major Depressive Disorder; AEDs—antiepileptic drugs.

**Table 2:** Comorbidity of MDE with AD in the group of epileptic patients (*n* = 96).

		Anxiety Disorders:								
		AD (+)			AD (−)			SUM		
Major depressive episode (MDE):		*N*			*N*			*N*		
		column%			column%			column%		
MDE (−)	*N*		8			67			75	
	row%	10.7%			89.3%			100%		
				50%			83.7%			78.1%
										
MDE (+)	*N*		8			13			21	
	row%	38.1%			61.9%			100%		
				50%			16.3%			11.9%
										
SUM	*N*		16			80			96	
	row%	16.7%			83.3%			100%		
				100%			100%			100%
										

test χ^2^ Pearson: χ^2^ = 8.8869, df = 1, *p* = 0.00287. AD—anxiety disorders.

## Data Availability

doi:10.1016/j.yebeh.2015.09.029, doi:10.1016/j.yebeh.2017.11.025.
